# The death rate of COVID-19 infection in different SARS-CoV-2 variants was related to C-reactive protein gene polymorphisms

**DOI:** 10.1038/s41598-024-51422-y

**Published:** 2024-01-06

**Authors:** Sahar Sadeghi Mofrad, Shayan Boozarjomehri Amnieh, Mohammad Reza Pakzad, Mina Zardadi, Morteza Ghazanfari Jajin, Enayat Anvari, Sina Moghaddam, Abolfazl Fateh

**Affiliations:** 1 Department of Microbiology, Islamic Azad University of Central Tehran Branch, Tehran, Iran; 2 Faculty of Veterinary Medicine, Tabriz Medical Science Branch, Islamic Azad University, Tabriz, Iran; 3 Department of Mycobacteriology and Pulmonary Research, Pasteur Institute of Iran, Tehran, Iran; 4 Clinical Research Development Unit, Shahid Mostafa Khomeini Hospital, Ilam University of Medical Science, Ilam, Iran; 5 Microbiology Research Center (MRC), Pasteur Institute of Iran, Tehran, Iran

**Keywords:** Microbiology, Virology, SARS-CoV-2

## Abstract

The serum level of C-reactive protein (CRP) is a significant independent risk factor for Coronavirus disease 2019 (COVID-19). A link was found between serum CRP and genetic diversity within the *CRP* gene in earlier research. This study examined whether *CRP* rs1205 and rs1800947 polymorphisms were associated with COVID-19 mortality among various severe acute respiratory syndrome Coronavirus 2 (SARS-CoV-2) variants. We genotyped *CRP* rs1205 and rs1800947 polymorphisms in 2023 deceased and 2307 recovered patients using the polymerase chain reaction-restriction fragment length polymorphism method. There was a significant difference between the recovered and the deceased patients in terms of the minor allele frequency of *CRP* rs1205 T and rs1800947 G. In all three variants, COVID-19 mortality rates were associated with *CRP* rs1800947 GG genotype. Furthermore, *CRP* rs1205 CC and rs1800947 GG genotypes showed higher CRP levels. It was found that the G-T haplotype was prevalent in all SARS-CoV-2 variants. The C–C and C–T haplotypes were statistically significant in Delta and Omicron BA.5 variants, respectively. In conclusion, polymorphisms within the *CRP* gene may relate to serum CRP levels and mortality among COVID-19 patients. In order to verify the utility of *CRP* polymorphism correlation in predicting COVID-19 mortality, a replication of these results is needed.

## Introduction

Since its initial appearance in Wuhan in December 2019, severe acute respiratory syndrome Coronavirus 2 (SARS-CoV-2) has caused more than 641 million cases of Coronavirus disease 2019 (COVID-19) and more than 6.6 million deaths. As well as being an excellent human pathogen, SARS-CoV-2 is a generalist when it comes to host tropism, infecting a wide variety of mammalian species^1^.

So far, the World Health Organization (WHO) has designated five SARS-CoV-2 variants as variants of concern (VOCs) because they have altered transmission or immune evasion and need special monitoring^2^. It was in the United Kingdom (UK) that the Alpha variant of SARS-CoV-2 was discovered during a period of low virus occurrence and exceptional SARS-CoV-2 surveillance. The Delta variant was discovered in India for the first time in April 2021. A fresh wave of epidemics in the UK soon followed, leading to the emergence of several sublineages, including AY.4.2, which was even more transmissible than Delta in its parental form. Compared to earlier VOCs, the Omicron variant complex has more mutations and a longer branch length^3,4^.

As a result of severe COVID-19, blood cell counts are altered, including an increase in neutrophils and leukocytes, a rise in C-reactive protein (CRP), and a drop in lymphocytes and platelets^5,6^.

The CRP concentration in the blood contributes to the physio-pathology of a variety of disorders and is a clear indicator of systemic inflammation. A high level of C-reactive protein is characteristic of inflammatory illnesses such as rheumatoid arthritis, cardiovascular diseases (CVD), and infections^7^. It has been observed that the SARS-CoV-2 virus increases plasma levels of CRP. When CRP levels rise, along with the start concentration, it indicates clinical deterioration, which is often accompanied by organ damage and an uncontrolled flare-up that promotes tissue fibrosis months after infection. As a result of CRP-mediated neutrophil macrophage activation, COVID-19 causes pulmonary fibrosis and eventual organ failure. COVID-19 infection can result in remarkably high plasma CRP levels^8–12^.

There are a number of single-nucleotide polymorphisms (SNPs) in the *CRP* gene that are also risk factors for diseases like cancer, arthritis, and diabetes^13^. In particular, *CRP* rs1205 (C/T) polymorphisms, located at the 3′ flanking region, were associated with CVD, type 2 diabetes, and all groups of neuropathies associated with metabolic disorders^14^.

A polymorphism in exon 2 of the *CRP* gene has been identified as + 1059 G > C (rs1800947). Due to the fact that it does not affect the amino acid composition of the protein structure, this polymorphism is a silent SNP. As previously reported, this SNP affects CRP protein levels, which influence type 2 diabetes and CVD^15^.

Due to the correlation between severity of COVID-19 disease and CRP levels, which indicate a more severe infection, as well as the correlation between elevated CRP levels in blood and SNPs in the CRP gene, this study examined two SNPs in the CRP gene (rs1205 and rs1800947) in order to determine how host genetic factors influence COVID-19 severity according to SARS-CoV-2 variations.

## Materials and methods

### Sample collection

The study was conducted out in accordance with the 1975 Declaration of Helsinki and any applicable local laws after receiving approval from the Ilam University of Medical Sciences Ethics Committee in Iran. All subjects provided their written informed consent.

According to the 1975 Declaration of Helsinki and any local laws applicable, the study was approved by the Ethics Committee at Ilam University of Medical Sciences in Iran (IR.MEDILAM. REC.I400.237). A written informed consent was provided by all subjects.

There were 4330 patients in this study with positive real-time reverse transcription-polymerase chain reaction tests for the SARS-CoV-2 Alpha, Delta, and Omicron BA.5 variants. Between September 2020 and May 2022, samples were collected from patients referred to a hospital in Ilam city, Iran.

Out of 13,450 cases, 4330 patients were recruited based on inclusion criteria, which include Kurdish nationality along with the same ethnicity, patients without prior infections with COVID-19 and vaccinations against SARS-CoV-2, and the absence of underlying comorbidities, including heart, lung, kidney, liver, cancer, autoimmune disease, diabetes, pregnancy, immunocompromised diseases, obesity, and diabetes.

In accordance with WHO guidelines, patients were divided into two groups based on their severity of disease and hospitalization requirements: 2307 outpatients (including those with mild or moderate symptoms) and 2023 inpatients (patients with severe or critical symptoms such as respiratory failure requiring mechanical ventilation, multiple organ dysfunction, distress, and SPO2 ≤ 88%)^16^. In the absence of healthy people without a history of COVID-19 to act as controls, recovered patients were used as controls and deceased patients were used as cases.

Upon entering the hospital, patient records of laboratory data were used to obtain hematological and biochemical parameters, including PCR cycle thresholds (Cts), C-reactive protein levels, lipid profiles, liver enzymes, kidney parameters, erythrocyte sedimentation rate (ESR), 25-hydroxyvitamin D levels, and fasting blood glucose levels (FBS).

### Genotyping the *CRP* rs1205 and rs1800947 polymorphisms

The total genomic DNA was extracted from 200 µL whole blood samples using a blood DNA extraction kit (Arman Gene Tajhiz Co.), according to manufacturer’s instructions. The extracted DNA was then analyzed for quality and purity using gel electrophoresis and NanoDrop spectrophotometers (Thermo Scientific, USA), respectively.

Genotyping of *CRP* rs1205 and rs1800947 polymorphisms was determined using the polymerase chain reaction–restriction fragment length polymorphism (PCR–RFLP). For *CRP* rs1205 polymorphism, the 227 bp fragment was amplified using primers f-5′-GGAGTGAGACATCTTCTTG-3′ and r-5′-CTTATAGACCTGGGCAGT-3′. The PCR products were digested with 1 U of *TaaI* (*HpyCH4III*) restriction enzyme (Thermo Fisher ScientificTM, USA). After digestion, the CC genotype had two fragments of 130 bp and 97 bp^17^.

The forward primer 5′-GATCTGTGTGTGATCTGAGAAACCTCT-3′ and the reverse primer 5′-GAGGATCCAGAGACAGAGACGTG-3′ were used to amplify 744 bp fragments for the analysis of the *CRP* rs1800947 polymorphism. PCR products were digested at 65 °C for 16 h with 1 U of *MaeIII* (Thermo Fisher ScientificTM, USA). The GG genotype produced three bands (310, 233, and 201 bp) after being digested by *MaeIII*, the CG genotype produced four bands (434, 310, 233, and 201 bp), while the CC genotype produced two bands (434 and 310 bp)^18^.

At least 10% of the samples were randomly genotyped with the Sanger sequencing technique on an ABI 3500 DX Genetic Analyzer (ABI, Thermo Fisher Scientific, MA, USA) to corroborate the results of the PCR. The raw data were analyzed with MEGA Version 11 (https://www.megasoftware.net/).

### Statistical analyses

Statistical analysis was performed using SPSS 22.0 software (SPSS. Inc, Chicago, IL, USA). We used the chi-square or Fisher exact test to compare discrete variables and the Hardy–Weinberg Equilibrium (HWE). Continuous variables were characterized by mean and standard deviation (SD), whereas categorical variables were described by number and percentage. The Mann–Whitney U test was used to examine continuously collected data. For two polymorphisms, haplotype frequencies, HWE, minor allele frequency (MAF), and all genetic models of inheritance (dominant, co-dominant, over-dominant, and recessive) using the Akaike Information Criterion (AIC) and the Bayesian Information Criterion (BIC) were evaluated by the SNPStats software (http://bioinfo.iconcologia.net/SNPStats). The odds ratios (ORs) and 95% confidence intervals (CIs) were analyzed to determine the strength of the correlation. All stated *P*-values are two-tailed, and 0.05 is used as the threshold for significance.

## Results

### The demographic properties of COVID-19 patients

A list of the characteristics of the COVID-19 study participants is shown in Table 1. Of the 4330 patients, 1395, 1425, and 1510 were Alpha, Delta, and Omicron BA.5 variants, respectively. Patients with the Alpha, Delta, and Omicron BA.5 variants had mean ages of 54.7 ± 12.5, 57.3 ± 12.1, and 52.4 ± 13.1, respectively (*P* < 0.001). In the Alpha, Delta, and Omicron BA.5 variants, the frequency of deceased patients was 632 (45.3%), 913 (64.1%), and 478 (31.7%), respectively. The number of males in the Alpha variant was 735 (52.7%), in the Delta variant was 760 (53.3%), and in the Omicron BA.5 variant was 765 (50.7%). The mean of qPCR Ct values (*P* < 0.001) and 25-hydroxy vitamin D (*P* < *0.*001) in the Delta variant was lower than other variants.

**Table 1:** Comparison of laboratory parameters between SARS-CoV-2 variants.

Variables	SARS-CoV-2 variants			*P*-value
	Alpha (n = 1395)	Delta (n = 1425)	Omicron BA.5 (n = 1510)	
Deceased/recovered patients	632/763 (45.3/54.7%)	913/512 (64.1/35.9%)	478/1032 (31.7/68.3%)	< 0.001*
Mean age ± SD	54.7 ± 12.5	57.3 ± 12.1	52.4 ± 13.1	< 0.001*
Gender (male/female)	735/660 (52.7/47.3%)	760/665 (53.3/46.7%)	765/745 (50.7/49.3%)	0.317
ALT, IU/L (mean ± SD) (Reference range: 5–40)	38.1 ± 24.7	40.1 ± 24.6	36.9 ± 24.7	< 0.001*
AST, IU/L (mean ± SD) (Reference range: 5–40)	33.2 ± 14.4	34.7 ± 14.0	34.4 ± 16.2	0.002*
ALP, IU/L (mean ± SD) (Reference range: up to 306)	184.5 ± 80.8	188.8 ± 76.5	186.0 ± 87.5	0.130
Cholesterol, mg/dL (mean ± SD) (Reference range: 50–200)	120.8 ± 39.1	120.6 ± 39.7	115.8 ± 33.1	0.007*
TG, mg/dL (mean ± SD) (Reference range: 60–165)	125.0 ± 53.0	120.7 ± 48.8	127.8 ± 58.1	0.084
LDL, mg/dL (mean ± SD) (Reference range: up to 150)	96.0 ± 48.3	84.1 ± 44.5	88.3 ± 47.6	< 0.001*
HDL, mg/dL (mean ± SD) (Reference range: > 40)	32.8 ± 11.6	32.0 ± 11.4	33.0 ± 11.4	0.016*
WBC, 10^9^/L (mean ± SD) (Reference range: 4000–10,000)	7645.4 ± 2786.5	7607.0 ± 2704.9	7685.1 ± 2931.2	0.922
CRP, mg/L (mean ± SD) (Reference range: < 10 mg/L Negative)	61.2 ± 22.0	64.3 ± 21.7	60.1 ± 21.2	< 0.001*
ESR, mm/1st h (mean ± SD) (Reference range: 0–15)	50.2 ± 16.2	52.7 ± 16.1	49.2 ± 15.7	< 0.001*
FBS, mg/dL (mean ± SD) (Reference range: 70–100)	108.0 ± 41.6	108.8 ± 42.5	106.1 ± 41.0	0.024*
Platelets × 1000/cumm (mean ± SD) (Reference range: 140,000–400,000)	184 ± 71	184 ± 74	184 ± 70	0.800
Uric acid, mg/dL (mean ± SD) (Reference range: 3.6–6.8)	4.8 ± 1.8	4.4 ± 1.7	5.1 ± 1.8	< 0.001*
Creatinine, mg/dL (mean ± SD) (Reference range: 0.6–1.4)	0.9 ± 0.3	1.0 ± 0.3	0.9 ± 0.3	< 0.001*
qPCR Ct value	20.2 ± 6.4	17.7 ± 6.2	21.2 ± 6.2	< 0.001*
25-hydroxy vitamin D, ng/mL (mean ± SD) (Sufficiency: 21–150)	31.8 ± 12.8	29.7 ± 12.3	33.6 ± 13.2	< 0.001*
*CRP* rs1205				< 0.001*
CC	389 (27.9%)	409 (28.7%)	630 (41.7%)	
CT	784 (56.2%)	723 (50.7%)	659 (43.7%)	
TT	222 (15.9%)	293 (20.6%)	221 (14.6%)	
*CRP* rs1800947				< 0.001*
CC	274 (19.6%)	291 (20.4%)	328 (21.7%)	
CG	937 (67.2%)	889 (62.4%)	1011 (66.5%)	
GG	184 (13.2%)	245 (17.2%)	171 (11.8%)	

ALT, alanine aminotransferase; AST, aspartate aminotransferase; ALP, alkaline phosphatase; TG, triglyceride; LDL, low density lipoprotein; HDL, high density lipoprotein; WBC, white blood cells; CRP, C-reactive protein; ESR, erythrocyte sedimentation rate; FBS, fasting blood glucose; SD, standard deviation; SARS-CoV-2: Severe Acute Respiratory Syndrome Coronavirus 2. *Statistically significant (< 0.05).

### Association between *CRP* rs1205 and rs1800947 polymorphisms and COVID-19 mortality based on SARS-CoV-2 variants

**Figure 1:**
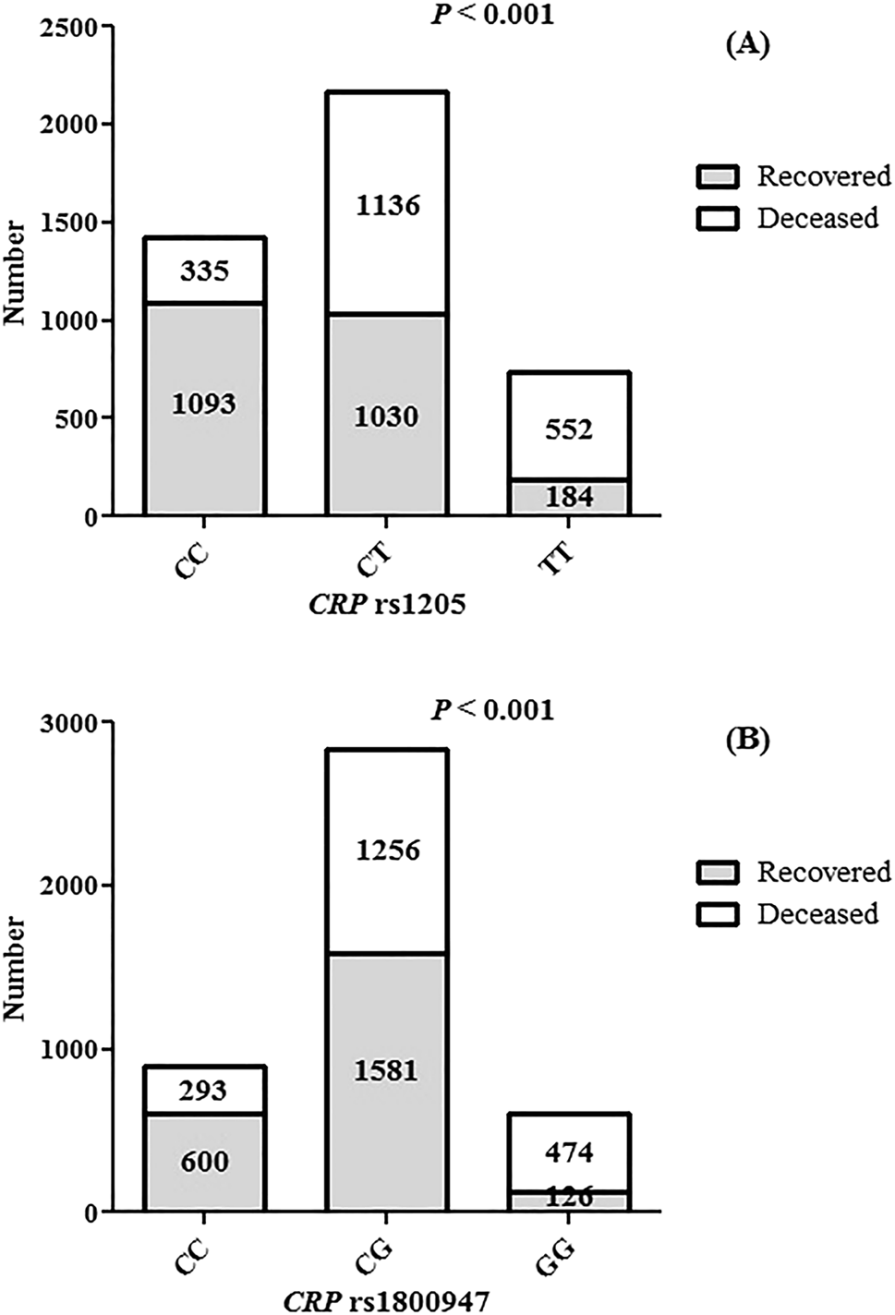
The frequencies of *CRP* rs1205 and rs1800847 on recovered and deceased patients.

Figure 1 illustrates that patients with the *CRP* rs1205 TT (*P* < 0.001) and rs1800947 GG (*P* < 0.001) genotypes had significantly higher COVID-19 death rates than patients with the other genotypes.

The findings of the examination of the patients’ *CRP* polymorphisms using an inheritance model are presented in Table 2.

**Table 2:** *CRP* SNPs association with COVID-19 mortality adjusted by SARS-CoV-2 variants.

*CRP* rs1205		Groups		OR (95% CI)	*P*-value	AIC	BIC
Model	Genotype	Recovered patients	Deceased patients				
Allele	C	3216 (70.0%)	1806 (45.0%)	–	–	–	–
	T	1398 (30.0%)	2240 (55.0%)	–	–	–	–
Codominant	C/C	1093 (47.4%)	335 (16.6%)	1.00	< 0.0001*	5124.9	5156.7
	C/T	1030 (44.6%)	1136 (56.1%)	3.54 (3.03–4.13)			
	T/T	184 (8.0%)	552 (27.3%)	9.74 (7.87–12.06)			
Dominant	C/C	1093 (47.4%)	335 (16.6%)	1.00	< 0.0001*	5237.5	5263.0
	C/T-T/T	1214 (52.6%)	1688 (83.4%)	4.49 (3.87–5.20)			
Recessive	C/C–C/T	2123 (92.0%)	1471 (72.7%)	1.00	< 0.0001*	5401.4	5426.9
	T/T	184 (8.0%)	552 (27.3%)	4.36 (3.62–5.25)			
Overdominant	C/C–T/T	1277 (55.4%)	887 (43.9%)	1.00	< 0.0001*	5627.8	5653.3
	C/T	1030 (44.6%)	1136 (56.1%)	1.55 (1.37–1.76)			
Minor allele frequency (T)		0.30	0.55	–	–	–	–
*CRP* rs1800947							
Allele	C	2781 (60.0%)	1842 (46.0%)	–	–	–	–
	G	1833 (40.0%)	2204 (54.0%)	–	–	–	–
Codominant	C/C	600 (26.0%)	293 (14.5%)	1.00	< 0.0001*	5354.2	5386.1
	C/G	1581 (68.5%)	1256 (62.1%)	1.70 (1.44–2.00)			
	G/G	126 (5.5%)	474 (23.4%)	7.96 (6.21–10.21)			
Dominant	C/C	600 (26.0%)	293 (14.5%)	1.00	< 0.0001*	5583.8	5609.3
	C/G-G/G	1707 (74.0%)	1730 (85.5%)	2.16 (1.84–2.54)			
Recessive	C/C–C/G	2181 (94.5%)	1549 (76.6%)	1.00	< 0.0001*	5393.2	5418.6
	G/G	126 (5.5%)	474 (23.4%)	5.29 (4.28–6.55)			
Overdominant	C/C-G/G	726 (31.5%)	767 (37.9%)	1.00	0.452	5660.9	5686.4
	C/G	1581 (68.5%)	1256 (62.1%)	0.80 (0.49–1.19)			
Minor allele frequency (G)		0.40	0.54	–	–	–	–

COVID-19, coronavirus disease; *CRP*, C-reactive protein; OR, Odds ratios; CI, confidence intervals; AIC, Akaike information criterion; BIC, Bayesian information criterion; OR, Odds ratios; CI, confidence intervals; *Statistically significant (< 0.05).

The codominant heredity models with the lowest BIC and AIC values were the best models for the both *CRP* polymorphisms. It was determined that *CRP* rs1205 TT genotype (*P* < 0.0001, OR 9.74, 95% CI 7.87–12.06) was linked to a greater chance to COVID-19 mortality. The *CRP* rs1800947 GG genotype (*P* < 0.0001, OR 7.96, 95% CI 6.21–10.21) was associated an increased chance to COVID-19 mortality.

In both recovered and deceased subjects, *CRP* polymorphisms were incompatible with HWE (*P* < 0.0001). The fact that these polymorphisms are not present in HWE is significant because it may assist to explain why the COVID-19 infection is linked to them.

### Correlation between the value of CRP and *CRP* gene polymorphisms

The mean plasma level of CRP in deceased patients (67.3 ± 21.3) was higher than recovered ones (57.0 ± 20.9), which was statistically significant (*P* < 0.001). Also, the mean plasma level of CRP in Alpha, Delta, and Omicron BA.5 variants was 61.2 ± 22.0, 64.3 ± 21.7, and 60.1 ± 21.2, respectively (*P* < 0.001).

There is significant difference in plasma level of CRP between different *CRP* rs1205 (*P* < 0.001) and *CRP* rs1800947 (*P* < 0.001) genotypes with recovered and deceased patients.

The mean plasma level of CRP in rs1205 CC, CT, and TT was 62.1 ± 21.4, 61.7 ± 21.8, and 61.2 ± 21.3, respectively (*P* = 0.070) and in *CRP* rs1800947 CC, CG, and GG genotypes was 60.3 ± 21.5, 61.7 ± 21.8, and 64.9 ± 21.3, respectively (*P* < 0.001). The highest amount of plasma level of CRP was found in *CRP* rs1205 CC and rs1800947 GG genotypes.

### Distributions of *CRP* polymorphisms among SARS-CoV-2 variants

Table 1 displays the frequency of the CC, CT, and TT genotypes of CRP rs1205 in the various SARS-CoV-2 variants. The CC, CT, and TT genotypes frequency in *CRP* rs1205 polymorphism in the Alpha variant were 389 (27.9%), 784 (56.2%), and 222 (15.9%), respectively. In the Delta variant, the frequency of CC was 409 (28.7%), CT was 723 (50.7%), and TT was 293 (20.6%). The rs1205 genotypes frequency in the Omicron BA.5 variant was 630 (41.7%), 659 (43.7%), and 221 (14.6%), respectively.

After adjusting the impact of *CRP* rs1205 polymorphism for SARS-CoV-2 variants, the COVID-19 mortality rate was correlated with *CRP* rs1205 TT and CT genotypes in the all three variants (Table 3).

**Table 3:** *CRP* SNPs association with SARS-CoV-2 variants.

Variants	rs1205 genotypes	Recovered patients	Deceased patients	OR (95% CI)
Alpha	C/C	292	97	1.00
	C/T	410	374	2.75 (2.10–3.59)
	T/T	61	161	7.95 (5.47–11.54)
Delta	C/C	288	121	1.00
	C/T	160	563	8.38 (6.36–11.03)
	T/T	64	229	8.52 (6.01–12.07)
Omicron BA.5	C/C	513	117	1.00
	C/T	460	199	1.90 (1.46–2.46)
	T/T	59	162	12.04 (8.40–17.25)

Variants	rs1800947 genotypes	Recovered patients	Deceased patients	OR (95% CI)
Alpha	C/C	186	88	1.00
	C/G	538	399	1.57 (1.18–2.08)
	G/G	39	145	7.86 (5.09–12.14)
Delta	C/C	198	93	1.00
	C/G	272	617	4.83 (3.63–6.42)
	G/G	42	203	10.29 (6.80–15.56)
Omicron BA.5	C/C	216	112	1.00
	C/G	771	240	–
	G/G	45	126	5.40 (3.58–8.14)

SARS-CoV-2, Severe Acute Respiratory Syndrome Coronavirus 2; *CRP*, C-reactive protein; OR: Odds ratios; CI, confidence intervals.

The CC, CG, and GG genotypes frequency in *CRP* rs1800947 polymorphism in the Alpha variant were 430 (42.1%), 937 (67.2%), and 184 (13.2%), respectively. In the Delta variant, the frequency of CC was 291 (20.4%), CG was 889 (62.4%), and GG was 245 (17.2%). The rs1800947 genotypes frequency in the Omicron BA.5 variant was 328 (21.7%), 1011 (66.5%), and 171 (11.8%), respectively (Table 1).

After adjusting the impact of *CRP* rs1800947 polymorphism for SARS-CoV-2 variants, the COVID-19 mortality rate was correlated with *CRP* rs1800947 GG in the all three variants and with *CRP* rs1800947 CG genotype in the Alpha and Delta variants (Table 3).

### Haplotype analysis

Our findings revealed that among all SARS-CoV-2 variations, the G-T haplotype was shown to be the prevalent form. The C–C haplotype was related to COVID-19 mortality in Delta (OR 1.64, 95% CI 1.20–2.24) variant. In the Omicron BA.5 variant, C–T haplotype (OR 3.32, 95%CI 1.68–6.57) was statistically significant (Table 4).

**Table 4:** SARS-CoV-2 variants and *CRP* SNPs haplotypes.

Haplotypes	Frequency	Alpha	Delta	Omicron
		OR (95% CI)	OR (95% CI)	OR (95% CI)
CC	0.5169	1.00	1.64 (1.20–2.24)	–
CG	0.0631	–	–	–
CT	0.017	–	–	3.32 (1.68–6.57)
GT	0.4031	2.62 (2.13–3.22)	6.66 (5.11–8.66)	1.34 (1.29–2.15)

SARS-CoV-2, Severe Acute Respiratory Syndrome Coronavirus 2; *CRP*, C-reactive protein; SNPs, single nucleotide polymorphisms; OR, Odds ratios; CI, confidence intervals.

## Discussion

The findings of this comprehensive study demonstrate considerable evidence that there are associations between two polymorphisms of the *CRP* gene (rs1205 and rs1800947) with COVID-19 mortality.

CRP levels were statistically significantly higher in deceased patients than in recovered patients, as shown in this study. Several studies have found that CRP can be used as a biomarker for COVID-19 infection severity and mortality^8,19^. Angiotensin II converting enzyme (ACE2) can produce CRP when it interacts with SARS‐CoV‐2^20^. The production of CRP by hepatocytes is stimulated by cytokines like interleukin‐6 (IL-6) and tumor necrosis factor-α (TNF-α) during COVID-19 development^21^. In one sense, CRP is responsible for activating the complement system, which contributes to inflammation. In contrast, severe COVID-19 patients will significantly damage their alveolar epithelial and endothelial barriers. The alveolar macrophages and epithelial cells are capable of producing a variety of cytokines and chemokines during an infection with SARS-CoV-2. In this phase, adaptive immunity is challenged by the significant decreases in lymphocytes and the T-cell-mediated immunosuppression. Therefore, in the context of COVID-19, uncontrolled SARS-CoV-2 infection could result in considerable macrophage infiltration, exacerbating acute lung damage^22,23^.

A number of SNPs are associated with plasma levels of CRP on the *CRP* gene. In addition to rs1205 and rs1800947 in the 3′- and 5′-flanking regions, rs1417938 is present in the intron, and rs1800947 appears in exon 2^24,25^.

Currently, this is the first study to evaluate the relationship between *CRP* rs1205 and rs1800947 and COVID-19 mortality. COVID-19 death rates were significantly higher in patients with the CRP rs1205 T allele. In all patients, the MAF (T allele) was 0.42, which was lower in recovered patients (0.30) than in died patients (0.55). In other regions, the T allele was found in 0.597 Asians, 0.609 East Asians, 0.342 South Asians, 0.545 other Asians, 0.332 Europeans, 0.333 Latin Americans, and 0.201 Africans, as reported in the NCBI dbSNP database (https://www.ncbi.nlm.nih.gov/snp/rs1205).

Several studies have demonstrated that CRP levels are functionally influenced by the rs1205 SNP. CRP is a major component of the innate immune system, so this SNP may have an impact on SARS-Co-2 pathogenesis. A genome-wide association meta-analysis found that an intron variant (rs67579710) was associated with COVID-19 hospitalizations in 24,741 cases and 2,835,201 controls. Due to its location within the thrombospondin-3 gene, this variant may affect thrombosis related to COVID-19 rather than inflammatory pathways^26,27^.

According to the current study, all three variants of *CRP* rs1205 T allele were correlated with COVID-19 mortality. Several studies have found that *CRP* rs1205 affects both clinical outcomes and vaccination outcomes. It has been suggested that the frequency of rs1205 T allele was significantly higher in patients with community-acquired pneumonia compared with healthy controls, and T allele was associated with an increased risk of infection. Furthermore, the rs1205 CT and TT genotypes were substantially more common in patients with severe community-acquired pneumonia than those with non-severe community-acquired pneumonia^18^.

As shown in our study, numerous studies with different diseases have shown that the rs1205 T allele is associated with lower serum CRP levels^18,28^. Since the rs1205 TT genotype results in lower serum CRP levels and is associated with lymph node metastasis in this form of cancer, rs1205 may be associated with thoracic esophageal squamous cell cancer, myocardial infarction, systemic lupus erythematosus and lupus nephritis^29,30^. In studies with higher baseline CRP levels, pre-eclampsia risk is positively correlated with *CRP* genotypes. In contrast, as in the current study, CRP genotypes (including the rs1205 T allele) associated with lower CRP levels and have been correlated with greater infectious load. There may have been balanced selection on CRP polymorphisms during evolution because of these disparate effects^18,31,32^.

In this study, patients with the CRP rs1800947 G allele had a significantly higher death rate from COVID-19. In all patients, the MAF (G allele) was 0.47, with recovery patients having a lower value (0.40) than dying patients having a higher value (0.54). A study in Iran found that the frequency of G-allele was 0.45, which is similar to what we found in our study^33^. According to NCBI dbSNP database (https://www.ncbi.nlm.nih.gov/snp/rs1800947), Asians and East Asians were most likely to have the G allele 0.036, South Asians 0.000, other Asians 0.040, Europeans 0.056, Latin Americans 0.024, and Africans 0.010, respectively.

According to our results, the COVID-19 mortality rate was correlated with *CRP* rs1800947 GG genotype in all three variants, as well as with CRP rs1800947 CG genotype in the Alpha and Delta variants of the gene.

There is a strong association between the *CRP* rs1800947 and *CRP* expression and it has been shown to be associated with heart disease, diabetes, and cancer^33–35^. In contrast to the results of this study, previous reports have demonstrated that C-allele carriers have lower plasma levels of CRP than GG genotype. Clinical diagnosis and severity assessment of community-acquired pneumonia and COVID-19 are based on serum CRP levels^19,36,37^. The high mortality rate in deceased people infected with COVID-19 with GG genotype could be due to their higher serum CRP levels.

There is no correlation between the majority of SNPs and diseases or functional issues. In contrast, if SNPs are located on genes or regulatory regions such as promoters, enhancers, they may influence the function of genes involved in disease mechanisms^36^. *CRP* rs1800947 may have different effects. Since the rs1800947 SNP is silent, the mechanism behind expressed *CRP* levels could be linkage disequilibrium between the rs1800947 polymorphism and other functional SNPs both inside and outside the CRP gene. There is also the possibility that this polymorphism alters the kinetics of CRP translation, causing variable levels of CRP throughout the body^38^.

In this study, several limitations were observed that should be considered in future studies. Because there were no healthy controls in this study, the outcomes were not compared with healthy controls. This study should be confirmed with more research on other ethnic groups in Iran, since this country has a variety of ethnicities.

In conclusion, this study showed that COVID-19 mortality rate was correlated with CRP rs1205 TT genotypes and *CRP* rs1800947 GG genotypes across all three variants. In addition, higher CRP levels were observed in individuals with the *CRP* rs1205 CC genotype and the *CRP* rs1800947 GG genotype. To verify these findings, further research should be conducted in other regions.

## Data Availability

All data that support all the experimental findings in this article is available in the paper.
